# An Artificial Neural Network Predicts Gender Differences of Motor and Non-Motor Symptoms of Patients with Advanced Parkinson’s Disease under Levodopa–Carbidopa Intestinal Gel

**DOI:** 10.3390/medicina60060873

**Published:** 2024-05-26

**Authors:** Anastasia Bougea, Tajedin Derikvand, Efthymia Efthimiopoulou

**Affiliations:** 1 1st Department of Neurology, Eginition Hospital, National and Kapodistrian University of Athens, 11572 Athens, Greece; faih.efthymiopoulou@gmail.com; 2 Department of Mathematics, Marvdasht Branch, Islamic Azad University, Marvdasht 73711-13119, Iran; ta.derikvand@iau.ac.ir

**Keywords:** advanced Parkinson’s disease, levodopa–carbidopaintestinal gel, Non-Motor Symptoms Questionnaire, NMS Questionnaire, Geriatric Depression Scale, Unified Parkinson’s Disease Rating Scale, Long Short-Term Memory–Recurrent Neural Network

## Abstract

*Background and Objectives*: Currently, no tool exists to predict clinical outcomes in patients with advanced Parkinson’s disease (PD) under levodopa–carbidopa intestinal gel (LCIG) treatment. The aim of this study was to develop a novel deep neural network model to predict the clinical outcomes of patients with advanced PD after two years of LCIG therapy. *Materials and Methods*: This was a longitudinal, 24-month observational study of 59 patients with advanced PD in a multicenter registry under LCIG treatment from September 2019 to September 2021, including 43 movement disorder centers. The data set includes 649 measurements of patients, which make an irregular time series, and they are turned into regular time series during the preprocessing phase. Motor status was assessed with the Unified Parkinson’s Disease Rating Scale (UPDRS) Parts III (off) and IV. The NMS was assessed by the NMS Questionnaire (NMSQ) and the Geriatric Depression Scale (GDS), the quality of life by PDQ-39, and severity by Hoehn and Yahr (HY). Multivariate linear regression, ARIMA, SARIMA, and Long Short-Term Memory–Recurrent NeuralNetwork (LSTM-RNN) models were used. *Results*: LCIG significantly improved dyskinesia duration and quality of life, with men experiencing a 19% and women a 10% greater improvement, respectively. Multivariate linear regression models showed that UPDRS-III decreased by 1.5 and 4.39 units per one-unit increase in the PDQ-39 and UPDRS-IV indexes, respectively. Although the ARIMA-(2,0,2) model is the best one with AIC criterion 101.8 and validation criteria MAE = 0.25, RMSE = 0.59, and RS = 0.49, it failed to predict PD patients’ features over a long period of time. Among all the time series models, the LSTM-RNN model predicts these clinical characteristics with the highest accuracy (MAE = 0.057, RMSE = 0.079, RS = 0.0053, mean square error = 0.0069). *Conclusions*: The LSTM-RNN model predicts, with the highest accuracy, gender-dependent clinical outcomes in patients with advanced PD after two years of LCIG therapy.

## 1. Introduction

Parkinson’s disease (PD) is a chronic neurodegenerative disease characterized by a complex range of motor and non-motor symptoms and has a major impact on the patient’s quality of life (QoL) [1,2]. Drug treatments for PD include levodopa in various concentrations with another compound (carbidopa) to improve the efficiency of levodopa and reduce side effects, such as nausea [1,2].Tremor and rigidity respond to levodopa treatment, but, particularly, when they occur later on in the disease, problems with standing, balance, and coordination are less likely to improve [1,2]. The most common side effects of levodopa are nausea, sleepiness, dizziness, and headache. More serious side effects can include confusion, hallucinations, delusions, agitation, and psychosis; these are more common in older people. Side effects can usually be avoided or minimized by starting with a low dose and increasing gradually.Dopamine agonists, oral or transdermal, may be used alone or as initial treatment forpeople with young-onset PD who are younger than 50 years old [1]. However, they are slightly less effective than levodopa and have more side effects, particularly, sedation, swelling of the legs, visual hallucinations, and impulse control disorders, such as compulsive gambling, eating, or shopping. MAO-B inhibitors can be used to treat “wearing off” in combination with levodopa or other antiparkinson drugs. Selegiline and rasagiline can also be taken alone as initial therapy for patients with mild motor symptoms of Parkinson’s disease. Side effects of MAO-B inhibitors can include nausea, headache, and difficulty falling asleep. When combined with levodopa, amantadine, an antiviral drug, may help to reduce dyskinesia in people with advanced PD. Possible side effects of amantadine include visual hallucinations and confusion, livedo reticularis (blotchy, purple-colored areas of skin usually found on the wrists and legs), and swelling of the ankles. The cholinesterase inhibitors (rivastigmine) may help to improve non-motor symptoms, such as dementia If adjusting medications does not improve non-motor symptoms (psychosis and hallucinations) adequately, an antipsychotic medication, such as quetiapine, may be used [2]. However, in the advanced stages of PD, patients may experience unsuccessful control of their motor complications through oral or transdermal levodopa medications. At this stage, there are several invasive therapeutic options (device-aided therapies) to be considered [3]. This lack of control could be explained by the inability to buffer exogenous dopamine that results from the denervation in the striatum, the short half-life of levodopa, and the delayed gastric emptying, which causes an unpredictable fluctuation of levodopa plasma.

The continuous delivery of levodopa as levodopa–carbidopa intestinal gel (LCIG) may overcome the above limitations improving motor/non-motor symptoms of patients with advanced PD and their QoL [4].However, not all patients with advanced PD are ideal candidates. To improve their selection analysis, deep knowledge of prognostic factors is essential. Moreover, longitudinal studies specifically addressing gender differences in LCIG treatments’ outcomes are lacking.

In this regard, artificial intelligence, including machine learning models (ML), could assist in treatment optimization for advanced PD [5,6,7,8]. One of the most advanced ML models to forecast time series is the Long Short-Term Memory (LSTM) Neural Network. The LSTM model is a very powerful tool for forecasting sequential issues in the field of public health [9]. Recently, an LSTM model predicted clinical factors and treatment outcomes [10,11]. In the same vein, the autoregressive moving average (ARIMA) and the seasonal ARIMA (SARIMA) models provide another predictive approach to time series forecasting of the spreading of various infectious diseases [12,13]. The results showed that combination models were better than single ARIMA models [14,15]. However, the accuracy of the combined model to directly predict the original sequence remains insufficient [16]. This study’s goal was to find the best deep neural network model by comparing it to the other models. This model would then be used to predict how both motor and non-motor symptoms would get worse in PD patients two years after LCIGimplementation.

## 2. Materials and Methods

### 2.1. Study Design

ForHealth S.A. is a Greek multicenter observational registry for patients with advanced PD under LCIG therapy, including 43 movement disorder centers in Greek cities (Athens, Thessaloniki, Patra, Ioannina, Chania, and Larisa). This was a longitudinal, 24-month, multicenter, observational study of 59 patients of ForHealth S.A. with advanced PD under LCIG treatment from September 2019 to September 2021. The preprocessing phase transforms the irregular time series of 649 patient measurements into a regular time series. Inclusion criteria were patients with advanced PD on stable LCIG regimens for the 24-month followup who were not receiving any other antiparkinsonian medication in the same period. The exclusion criteria included dementia and major depression. The following data were collected: demographics, disease duration, and motor and non-motor clinical measures at ten time points (T1 = 1 month, T2 = 3 months, T3 = 6 months, T4 = 9 months, T5 = 12 months, T6 = 16 months, T7 = 18 months, T8 = 20 months, T9 = 22 months, and T10 = 24 months).

We used a portable pump (CADD-Legacy^®^, Duodopa CE 0473) [4] to give LCIG (20 mg/mL) over 16 h through a PEG-J (percutaneous endoscopic gastrostomy tube with jejunal extension). The LCIG treatment consisted of three individually adjusted doses: the morning bolus dose, typically 5–10 mL [100–200 mg levodopa], the continuous maintenance dose, typically 2–6 mL/h [40–120 mg levodopa], and additional bolus doses, and each was individually adjusted.

Motor status was assessed with the Unified Parkinson’s Disease Rating Scale Part III (UPDRS-III) in the offstage. Hours of “off” and dyskinesia duration were assessed with the Unified Parkinson’s Disease Rating Scale, Part IV (UPDRS-IV) [17]. The Non-Motor Symptoms Questionnaire (NMSQ) [18] and the Geriatric Depression Scale (GDS) evaluated 17 non-motor symptoms [19]. We assessed the severity of PD using the Hoehn and Yahr (HY) [20] and the quality of life using the PD questionnaire (PDQ-39) [21]. All procedures performed in studies involving human participants were in accordance with the ethical standards of the institutional and/or national research committee and with the 1964 Helsinki Declaration and its later amendments or comparable ethical standards. The ethics committee of AbbVie Greece approved the study protocol, with approval No. 1041/20-6-2019. We reported this study in accordance with the Strengthening the Reporting of Observational Studies in Epidemiology (STROBE) guideline (Table S1 in Supplementary Materials).

### 2.2. Statistical Analysis

The patient’s demographics were summarized using descriptive statistics. Quantitative data were expressed as mean  ±  standard deviation (SD). In the data preprocessing phase, some missing data were computed using their column’s mean, and a one-dimensional wavelet transform was used to remove possible noise in all columns. Shapiro–Wilk and Kolmogorov–Smirnov normality tests confirmed the normal distribution of all features in the men and women subgroups before and after LCIG treatment. Independent sample *t*-tests and paired sample *t*-tests were used to compare the LCIG effects on QL in PD patients. A multi-regression model was fitted for the measured clinical characteristics of men and women separately, and the possible synergistic effects of the features were evaluated. The spline interpolation method transformed irregular time series into regular ones. We derived the best seasonal ARIMA model for the time series and forecasted clinical measures for the ten subsequent time points. We presented a machine learning method for patient classification, which allowed us to determine the class or grade of the new patients based on their known PD characteristics. The moving average (MA) term in a time series model is a past error multiplied by a coefficient. The LSTM model was used to predict future values based on previous, sequential data. The R language and environment were used for statistical computing and graphics. Statistical significance was set at *p* = 0.05.

## 3. Results

This study included fifty-nine patients. At baseline, the mean age was 71 ± 10.18 years, and 47.6% were men. The mean disease duration was 14.28 ± 5.64 years. At baseline, clinical characteristics were the following: dyskinesia duration (2.44 ± 0.61), HY (2.05 ± 0.2), UPDRS-III (off) (30.50 ± 4.15), UPDRS-IV (3.13 ± 1.02), GDS (7.48 ± 2.42), NMSQ (10.09 ± 0.97), and PDQ-39 (37.50 ± 0.79). Table 1 summarizes the main demographic and clinical characteristics.

Our data set includes seven clinical features of PD patients at eleven different time steps, before and after LCIG implementation (Table 1). Men and women improve all features, but both groups significantly decrease the duration of dyskinesia, and the variation of UPDRS-III is sexdependent.

**Figure 1:**
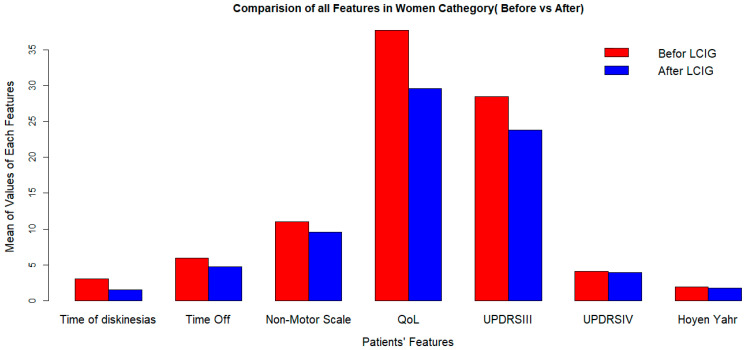
Clinical outcomes after LCIG implementation in women at 2-years followup. LCIG: levodopa–carbidopa infusion gel, QoL: quality of life, UPDRSIII:Unified Parkinson’s Disease Rating Scale III.

**Figure 2:**
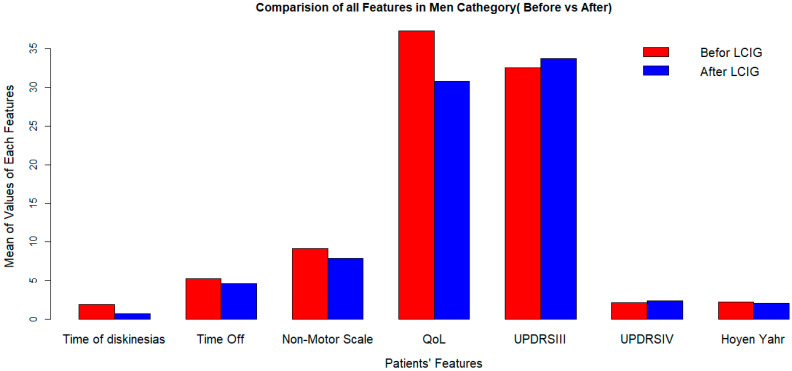
Clinical outcomes after LCIG initiation in men at 2-years followup. LCIG: levodopa–carbidopa infusion gel, QoL: quality of life, UPDRSIII: Unified Parkinson’s Disease Rating Scale III.

LCIG significantly improves quality of life, but its effects on dyskinesia duration and UPDRS-III (off) score vary by sex (Figure 1 and Figure 2).

**Figure 3:**
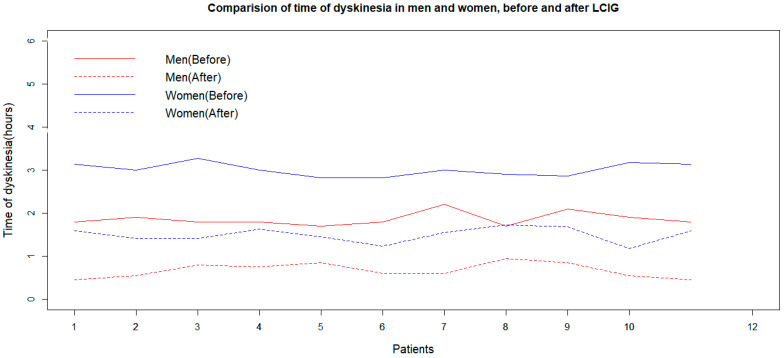
Effects of LCIG treatment on dyskinesia duration in men and women (before and 2 years after the initiation of LCIG treatment). LCIG: levodopa–carbidopa intestinal gel.

The Shapiro–Wilk normality test shows that the duration of dyskinesia was normally distributed in the women and men groups before and after LCIG treatment (*p*-values of 0.448, 0.445, 0.049, and 0.311, respectively). The F-test and box plot of data prove the equality of variances of dyskinesia duration values for men and women before and after LCIG (*p*-values of the F-tests were 0.653, 0.750, 0.951, and 0.943, respectively). Both women and men experience a reduction in dyskinesia duration after LCIG treatment, as indicated by the *p*-values of a paired sample *t*-test. Two independent sample *t*-tests showed that the effect of LCIG treatment on dyskinesia duration is better in men compared to women (*p* = 0.002, Figure 3).

### 3.1. Multivariate Linear Regression Model

The UPDRS-III (off) score was considered the response variable, while NMSQ, PDQ-39, UPDRS-IV, HY scores, and dyskinesia duration at the baseline were predictors. The slopes and intercepts are 82, 0.20, −1.5, −4.39, 2.63, and 4.82. For every unit increase in the PDQ-39 and UPDRS-IV indexes, the UPDRS-III score decreased by 1.5 and 4.39 units, respectively. Additionally, the HY score and dyskinesia duration increased the UPDRS-III (off) score by 2.63 and 4.82 units, respectively.

Dyskinesia duration is considered the response variable, while NMSQ, PDQ-39, UPDRS-IV, HY, and UPDRS-III (off) scores at the baseline are predictors. The slopes and intercepts are −0.45, −0.07, 0.09, 0.51, −0.81, and 0.009, respectively. The UPDRS-IV index increases dyskinesia duration by 0.51 units for every unit increase. The HY also decreases dyskinesia duration by 0.81 units for every unit increase. The linear model allows for the omission of other features. Note that this regression model explored a linear relationship between the response variable of dyskinesia duration and the predictors of NMSQ, PDQ-39, UPS-IV, and HY at the baseline, allowing for the neglect of the most significant coefficient associated with PDQ-39 and the other scores. Dyskinesia duration increased by one unit against half of a unit of the PDQ-39 increment. In another linear regression model, PDQ-39 was considered the dependent variable, while UPDRS-IV, UPDRS-III, and HY were the predictors. Therein, the coefficient values are 0.467, −0.0476, and 2.489, respectively. PDQ-39 displays a strong linear relationship to the HY scale. In another model, the NMSQ score was supposed to be the response variable for the predictors of the UPDRS-III (off), UPDRS-IV, and HY scores. The coefficients were 0.006, 0.786, and −0.699, respectively. This asserts that NMSQ scores are linearly dependent on UPDRS-IV and HY scores.

### 3.2. Mathematical Modeling

We present two types of models from two different approaches to predict the patients’ features with an acceptable horizon. First, among the time series models, we introduce the best model and estimate the model’s most accurate parameters for this purpose. The Akaike information criterion (AIC) will be used to find optimum parameters in the sequel. Second, a recurrent neural network model will be used with a different approach to forecasting the future values of these features. The MAE, RMSE, and RS criteria will determine the accuracy of the model. After the preprocessing phase, since the data are not equidistant, cubicspline interpolation was used to convert this irregular time series to a regular one. The cycle of this time series is 31 days in a month, and the data are distributed across the spectrum.

### 3.3. Time Series Models

**Figure 4:**
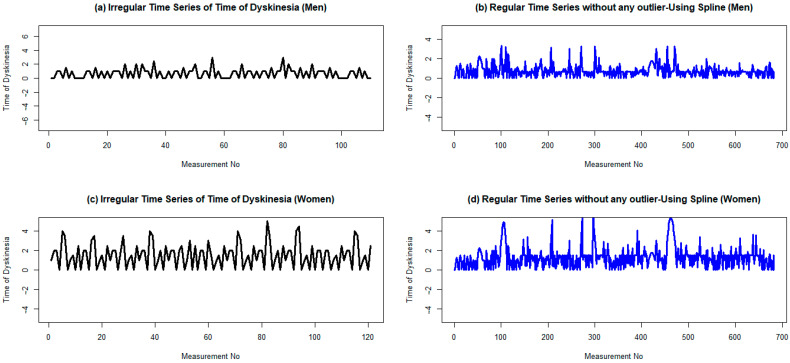
Regular and irregular time series of dyskinesia duration for men and women two years after the initiation of LCIG treatmentusing cubic spline. (**a**,**b**) Are plots ofthe irregular time series with a frequency of 1 dyskinesia duration for men and women, respectively. (**c**,**d**) Are plots of their corresponding irregular time series without any outlier data. To omit possible outlier data, the lower and upper bounds are considered as 1.5 times the IQR (interquartile range), less than the first quartile, and 1.5 times the IQR, greater than the second quartile, respectively.

The augmented Dickey–Fuller test (ADF test) confirms that the time series is stationary, with *p*-values of less than 0.01 and an ADF value of –6.18, which is less than the critical value of –2.567. Moreover, the Kwiatkowski–Phillips–Schmidt–Shin test (KPSS test) confirms the same result. The means of the two parts are 0.77 and 0.72 after partitioning into a couple of groups, with variances of 0.40 and 0.27, respectively. This confirms the stationary assumption of the time series. Finally, the plot of the regular time series of dyskinesia duration of male patients after LCIG confirms that the produced signal is stationary (Figure 4).

**Figure 5:**
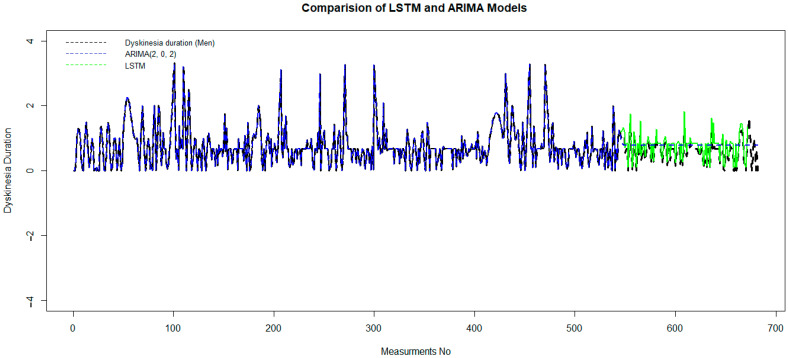
Comparison of the LSTM and ARIMA models to predictdyskinesia duration for patients under LCIG. LCIG: levodopa–carbidopa intestinal gel.

The pattern of both the autocorrelation function (ACF) and the partial autocorrelation function (PACF) in the tail of the ARIMA model [combine two models, Auto Regression (AR) and moving average (MA)] is better than that of other time series models. p = 2 and q = 2 are the best-estimated parameters of the model, where p indicates the number of autoregressive terms (lags of the stationarized series), and q is the trend-moving average order. We obtain these optimum parameters based on a minimum Akaike information criterion (AIC), which, in this case, equals 101.8. This model has been trained on 80% of the data set, and it has been tested in the second part of that. The validation criteria, MAE = 0.25, RMSE = 0.59, and RS = 0.49, measure the accuracy of the model. Although the ARIMA model is good for short-horizon prediction, it fails to fit as well as the LSTM model, which is significant for long-horizon prediction (Figure 5).

### 3.4. LSTM Network

In the building model step, the sequential model is created of two LSTM and dens layers for data according to fifty epochs. The input data of the LSTM network is in the form of three-dimensional arrays. This model was developed by building the data in eleventime steps (time steps) and converting them into an array with one feature in each input.

In order to evaluate and obtain the quality of each model, the mean square error value is calculated on 20% of the data set as validation data.

The Relu activation function and Adam optimizer with a learning rate of 0.01 are used to obtain the output of the desired layer (Table S2).

After preparing the data set and removing outlier data with a suitable wavelet transform, an LSTM algorithm has been trained on 80% of the data set, and it has been tested in the second part. In Figure 5, green and blue curvatures display the test and predicted data of dyskinesia duration using LSTM and ARIMA (2, 0, 2). 

Although it is obvious that black and green dashed lines coincided well, in order to measure the accuracy of the LSTM model, the error value is computed by mean square error. The MAE = 0.057, RMSE = 0.079, and RS = 0.0053 show the accuracy of this model. This demonstrates that the performance of the LSTM model is much better than the ARIMA model.

## 4. Discussion

This study presents simple visualization tools that show trends evolving or repeating over time to advanced Μl to discover the specific structure of time series related to the PD patients’ data set. According to this harmonic analysis, LCIG therapy can significantly improve the duration of dyskinesia. The UPDRS-III (off) score variation under LCIG therapy is sexdependent. Different time series models are fitted to predict the time of dyskinesia after LCIG therapy. Among these models, the ARIMA-(2,0,2) model is the best one and has an AIC criterion of 101.8, and its validation criteria are MAE = 0.25, RMSE = 0.59, and RS = 0.49. However, the traditional time series models fail to predict the values of PD patients’ features over a long period of time. An RNN model was adjusted to predict these features. To our knowledge, this is the first attempt to predict the motor and non-motor features of patients with advanced PD under LCIG using a deep neural network model, such as the RNN-LSTM model. The accuracy criteria, MAE = 0.057, RMSE = 0.079, and RS = 0.0053, showed high validation to predict PD features in the test part of the data for the proposed LSTM model.

A novel finding of this study was the better effect of LCIG on the time of dyskinesia in men than in women. In line with previous studies, women are more likely than men to develop dyskinesia before the LCIG treatment [22,23,24,25]. Although clinical trials with LCIG were successful in reducing motor symptoms, including dyskinesia, sex differences were not fully assessed [22,23,24,25,26,27,28,29,30]. The etiology of the sex effect is not clear. It has been suggested that hormonal status may modify sensitivity to levodopa [23] and that lower body weight may lead to greater levodopa exposure [24]. Higher LEDD (≥2000 mg/day) was required in men compared to women [25]. A higher discontinuation rate due to non-procedure or device-associated adverse effects was noted in the ≥2000 mg/day group [26]. Future studies will be useful for decisionmaking with regard to LCIG therapy for individual patients.

In the present study, LCIG treatment significantly improved the QoL assessed by PDQ-39, which is in agreement with previous reports [27,28,29]. Specifically, UPDRS-III decreased by 1.5 and 4.39 units per one unit increase in PDQ-39, which is similar to the findings of other studies [27,31,32,33,34]. We observed UPDRS-IV, UPDRS-III, and HY scores as predictors for the PDQ-39 score. HY showed a strong association with PDQ-39. A possible explanation could be that a poor QoL reflects the greater severity of the disease later in the disease’s course.

Importantly, UPDRS-III (off), UPDRS-IV, and HY scores were predictors for the total score of the NMSQ. However, the HY scale does not necessarily reflect therapy-related improvements in NMS, which are not well captured by this staging system. On the other hand, the burden of NMS at the baseline predicts improvement in the QoL in patients treated with LCIG. However, these studies were singlecentered and not longitudinal, including non-naïve PD patients.

### 4.1. Limitations and Strengths

Our study also has some limitations, such as a lack of a control group. This is a direct measurement and analysis of data, and the validity of the method and its results were asserted by the performance of the method on the test data set, not in comparison with a control group. PD is a non-homogeneous disease with many subtypes. We did not explore subtypes of PD or whether our artificial neural network worked equally well with all subtypes. We validated both the progression analysis and preclinical diagnosis in a limited number of participants, which is another limitation of our study. In this study, we measured seven features for each of fifty-two patients eleven times. Therefore, our data set includes 572 measurements, which isacceptable considering the limitations and cost of patient monitoring. We have also tested the model across institutions, using independent datasets. Future studies should expand the diversity of datasets and institutions. However, further analysis is necessary to fully understand the mechanisms that lead to the development and progression of motor and non-motor symptoms in advanced PD.

Nonetheless, the strengths of our study include a very thorough assessment, a longitudinal follow-up design, and extensive clinical and demographic information recorded at ten time points. By tracking the progression of advanced PD under LCIG monotherapy and including only Part III scores in the off-medicationstate, potential confounding from different medication effects at baseline assessments has been reduced.

### 4.2. Future Research Directions/Possible Applications of the Research

Using LSTM, timeseries forecasting models can predicttreatment outcomes based on sequential data [11,35]. This provides greater accuracy for ML methods. LSTM-based recurrent neural networks could be a powerful approach to learning from sequential data. The LSTM cell enhances performance by adding long-term memory, enabling the learning of more parameters. This makes it the most powerful recurrent neural network for forecasting, especially in the presence of a longer-term trend in the data set. A previous study analyzed clinical, genetic, imaging, and cerebrospinal fluid markers to predict the onset of symptomatic therapy and motor progression based on UPDRS III [36]. This study is a small, but determined, step in attaining a better view and knowledge regarding the more advanced means and their usage in ML applications. We have only used the data of the training group to find the desired model, and then we have used the test data to validate the model. Each feature has been measured eleven times; hence, we have eleven sets of values for all features. Consequently, this code has been run eleven times, and the same results are achieved, which proves the validity and accuracy of the model. The mean squared error of 0.0069 is a suitable criterion for evaluating the validity of the presented model.

We suggested that future timeseries forecasting models should predict the development of mental changes, depression with suicidal tendencies, and other serious mental changes inpatients under LCIG. By monitoring these patients regularly for the development of impulse control disorders, for example, Dopamine Dysregulation Syndrome (DDS), it is of great importance to confirm the effectiveness of the LCIG therapy.Future timeseries forecasting models should also incorporate fluid and neuroimaging biomarkers (such as a- synuclein, microRNA, neurofilaments, MRI, DatSCAN). These applications of the research will demonstrate ML potential for supporting a new generation of adaptive LCIG therapy, with better management of motor and non-motor symptoms, resulting in more efficient and personalized patient-tailored treatments.

## 5. Conclusions

In the new eraofartificial intelligence, this RNN-LSTM model could potentially be used as an additional tool in health decisionmaking to predict dyskinesia duration and quality of life in PD patients after 2-year LCIG therapy. We need more longitudinal studies that combine fluid and imaging biomarkers to accurately select ideal candidates for LCIG therapy.

## Figures and Tables

**Table 1:** Clinical features of patients with PD before and after using LCIG treatment.

	Men (before LCIG Treatment)	Men (after LCIG Treatment)	Women (before LCIG Treatment)	Women (after LCIG Treatment)
Age	70 ± 12.08	-	73.45 ± 8.35	-
PD Duration	14.3 ± 4.83	-	14.27 ± 6.52	-
GDS	8.18 ± 2.09	-	6.7 ± 2.62	-
Dyskinesia Duration	1.86 ± 0.16	0.67 ± 0.17	3.01 ± 0.15	1.49 ± 0.18
“Off” Duration	5.26 ± 0.25	4.57 ± 0.43	5.94 ± 0.25	4.73 ± 0.52
NMSQ	9.15 ± 0.24	7.89 ± 0.84	11.01 ± 0.15	9.53 ± 1.27
UPDRS-III (state off)	32.55 ± 4.78	33.76 ± 3.43	28.45 ± 2.03	23.79 ± 4.78
UPDRS-IV	2.14 ± 0.12	2.34 ± 0.93	4.11 ± 0.25	3.90 ± 0.09
HY	2.21± 0.18	2.04 ± 0.08	1.88 ± 0.10	1.75 ± 0.20
PDQ-39	37.31 ± 0.94	30.77 ± 1.30	37.67 ± 0.59	29.61 ± 2.19

GDS, Geriatric Depression Scale; HY, Hoehn and Yahr; LCIG, levodopa–carbidopa intestinal gel; NMSQ, Non-Motor Symptoms Questionnaire; PDQ, Parkinson’s disease; PDQ-39, Parkinson’s Disease Questionnaire; UPDRS III, Unified Parkinson’s Disease Rating Scale Part III.

## Data Availability

A preprint of this study is available at https://www.medrxiv.Org/content/10.1101/2023.06.26.23291833v1 (accessed on 1 January 2024)

## Supplementary Materials

The following supporting information can be downloaded at https://www.mdpi.com/article/10.3390/medicina60060873/s1, Table S1: STROBE Statement—checklist of items that should be included in reports of cohort studies; Table S2: Relu activation function for layers and outputs.
