# Supplementary material for: An Artificial Neural Network Predicts Gender Differences of Motor and Non-Motor Symptoms of Patients with Advanced Parkinson’s Disease under Levodopa–Carbidopa Intestinal Gel
Source: Medicina (Kaunas). 2024 May 26;60(6):873. doi: 10.3390/medicina60060873 (PMC11206121; doi:10.3390/medicina60060873)
Supplement: Supplementary file 1 [file medicina-60-00873-s001.zip › medicina-3017706-supplementary.pdf]

Table S1. STROBE Statement—Checklist of items that should be included in reports of ***cohort studies***

	Item No	Recommendation
**Title and abstract**	1	(*a*) Indicate the study's design with a commonly used term in the title or the abstract page1
		(*b*) Provide in the abstract an informative and balanced summary of what was done and what was found page4
**Introduction**		
Background/rationale	2	Explain the scientific background and rationale for the investigation being reported page5
Objectives	3	State specific objectives, including any prespecified hypotheses page6
**Methods**		
Study design	4	Present key elements of study design early in the paper page6
Setting	5	Describe the setting, locations, and relevant dates, including periods of recruitment, exposure, follow-up, and data collection page6
Participants	6	(*a*) Give the eligibility criteria, and the sources and methods of selection of participants. Describe methods of follow-up page6
		(*b*) For matched studies, give matching criteria and number of exposed and unexposed
Variables	7	Clearly define all outcomes, exposures, predictors, potential confounders, and effect modifiers. Give diagnostic criteria, if applicable page6
Data sources/ measurement	8*	For each variable of interest, give sources of data and details of methods of assessment (measurement). Describe comparability of assessment methods if there is more than one group page6
Bias	9	Describe any efforts to address potential sources of bias page6
Study size	10	Explain how the study size was arrived at page6
Quantitative variables	11	Explain how quantitative variables were handled in the analyses. If applicable, describe which groupings were chosen and why page7
Statistical methods	12	(*a*) Describe all statistical methods, including those used to control for confounding page 7-8
		(*b*) Describe any methods used to examine subgroups and interactions page 7-8
		(*c*) Explain how missing data were addressed page 7-8
		(*d*) If applicable, explain how loss to follow-up was addressed page 7-8
		(*e*) Describe any sensitivity analyses page 7-8

**Results**		
Participants	13*	(a) Report numbers of individuals at each stage of study—eg numbers potentially eligible, examined for eligibility, confirmed eligible, included in the study, completing follow-up, and analysed page 8
		(b) Give reasons for non-participation at each stage
		(c) Consider use of a flow diagram
Descriptive data	14*	(a) Give characteristics of study participants (eg demographic, clinical, social) and information on exposures and potential confounders page 8
		(b) Indicate number of participants with missing data for each variable of interest
		(c) Summarise follow-up time (eg, average and total amount)
Outcome data	15*	Report numbers of outcome events or summary measures over time page 8-11
Main results	16	(*a*) Give unadjusted estimates and, if applicable, confounder-adjusted estimates and their precision (eg, 95% confidence interval). Make clear which confounders were adjusted for and why they were included NOT APLICABLE
		(*b*) Report category boundaries when continuous variables were categorized NOT APLICABLE
		(*c*) If relevant, consider translating estimates of relative risk into absolute risk for a meaningful time period NOT APLICABLE
Other analyses	17	Report other analyses done—eg analyses of subgroups and interactions, and sensitivity analyses page 8-11
**Discussion**		
Key results	18	Summarise key results with reference to study objectives page 12
Limitations	19	Discuss limitations of the study, taking into account sources of potential bias or imprecision. Discuss both direction and magnitude of any potential bias page 14
Interpretation	20	Give a cautious overall interpretation of results considering objectives, limitations, multiplicity of analyses, results from similar studies, and other relevant evidence page 12-13
Generalisability	21	Discuss the generalisability (external validity) of the study results page 14
**Other information**		
Funding	22	Give the source of funding and the role of the funders for the present study and, if applicable, for the original study on which the present article is based page 3

*Give information separately for exposed and unexposed groups.

Note: An Explanation and Elaboration article discusses each checklist item and gives methodological background and published examples of transparent reporting. The STROBE checklist is best used in conjunction with this article (freely available on the Web sites of PLoS Medicine at http://www.plosmedicine.org/, Annals of Internal Medicine at http://www.annals.org/, and Epidemiology at http://www.epidem.com/). Information on the STROBE Initiative is available at http://www.strobe-statement.org.

Table S2. *Relu activation function for layers and outputs*

Layer (type)	Output Shape	Param #
1stm_1(LSTM)	(1, 11, 50)	10400
dropout_1 (Dropout)	(1, 11, 50)	0
1stm (Dropout)	(1, 11, 50)	20200
dropout (Dropout)	(1, 11, 50)	0
time_distributed (TimeDistributed)	(1, 11, 1)	51
Total params: 30651 (119.73 KB)<br>Trainable params: 30651 (119.73 KB)<br>Non-Trainable params: 0 (0.00 KB)		

LSTM: Long short-term memory
